# Recent Advancements in Cyclodextrin-Based Adsorbents for the Removal of Hazardous Pollutants from Waters

**DOI:** 10.3390/polym14122341

**Published:** 2022-06-09

**Authors:** Shan E. Zehra Syeda, Dominika Nowacka, Muhammad Shahzeb Khan, Anna Maria Skwierawska

**Affiliations:** Department of Chemistry and Technology of Functional Materials, Faculty of Chemistry, Gdańsk University of Technology, 11/12 Narutowicza Street, 80-233 Gdańsk, Poland

**Keywords:** cyclodextrin-based adsorbents, water pollution, heavy metals, dyes, organic pollutants

## Abstract

Water is an essential substance for the survival on Earth of all living organisms. However, population growth has disturbed the natural phenomenon of living, due to industrial growth to meet ever expanding demands, and, hence, an exponential increase in environmental pollution has been reported in the last few decades. Moreover, water pollution has drawn major attention for its adverse effects on human health and the ecosystem. Various techniques have been used to treat wastewater, including biofiltration, activated sludge, membrane filtration, active oxidation process and adsorption. Among the mentioned, the last method is becoming very popular. Moreover, among the sorbents, those based on cyclodextrin have gained worldwide attention due to their excellent properties. This review article overviewed recent contributions related to the synthesis of Cyclodextrin (CD)-based adsorbents to treat wastewater, and their applications, especially for the removal of heavy metals, dyes, and organic pollutants (pharmaceuticals and endocrine disruptor chemicals). Furthermore, new adsorption trends and trials related to CD-based materials are also discussed regarding their regenerative potential. Finally, this review could be an inspiration for new research and could also anticipate future directions and challenges associated with CD-based adsorbents.

## 1. Introduction

Water is a vital substance for all living organisms [1], and it plays an essential role in medical, energy and agricultural fields [2]. Through natural and anthropogenic activities, there has been an increase in contaminants and water bodies have deteriorated. Heavy metals, pesticides, dyes, pharmaceutical waste, and other emerging pollutants, have become a burden on the ecological environment [3,4,5]. These harmful contaminants could be bio-accumulated in the food chain and cause adverse human health impacts [6]. Pharmaceutical residues in water have become life-threatening, and directly affect the ecosystem and human health [7]. They get released in an aqueous environment through treatment plants and industrial effluent. Removal of toxic xenobiotic pollutants has become a growing concern in water treatment. Moreover, the concentration of pharmaceutical contaminants in water resources is directly connected to human consumption patterns, population growth, industrial activities, and wastewater treatment [8]. Recently, low concentrations have been found to possess higher toxicity and their removal from the aqueous environment has become a matter of urgency. To remove such pollutants, some biological, chemical, and physical approaches, including adsorption, filtration, coagulation, flocculation, and precipitation, have been utilized [9,10,11,12,13].

Furthermore, it is reported in many studies that pesticides are toxic substances for the environment and human health [14]. The frequent use of pesticides poses a threat to ecosystems and directly affects wildlife, birds, domestic animals, livestock, fish, and soil [15]. In addition, many pesticides could be stable over time, transported in water and air, and ultimately contaminate areas far from their point of origin [16]. The enormous increase in the world population from 1900 to 2000 (1.5 billion to 6.9 billion) has also increased pressure on food demand; hence, the usage of pesticides has increased exponentially to meet production and economic profit levels [17]. Therefore, the removal of pesticides is a crucial research domain [18,19], so, various treatment techniques have been used to remove them. Currently, advanced oxidation processes (AOPs) [20], photochemical degradation [21], chlorination [22], and adsorption are favored techniques.

Dyes also pose considerable concerns to the environment and ecosystem due to their high toxic levels, and some of the colorants have products after their reactions. They may cause adverse impacts on aquatic life by disrupting biological processes [23,24]. As a result, heavy metals are more complicated to remove than the dyes, and thus they accumulate in water and soil [25,26]. They could also cause toxicosis in humans by contaminating water and crops [27,28]. Researchers pay special attention to the heavy metals present in ground and surface water [29]. Heavy metals, such as As(III), As(V), Cu(II), Ni(II), Co(II), Cd(II), Pb(II), Cr(III), etc., can be discharged directly and indirectly into water bodies [30,31,32]. Since they are carcinogenic and non-biodegradable, they can affect the growth of biological tissues in living organisms [33,34]. 

People have been so focused on industrial development that they stopped paying attention to the environment, and hence pollutants became stronger, more resistant, and progressively deposited in organisms [35,36]. Consequently, researchers have fixated on the research domain related to developing cost-effective removal techniques for heavy metals, organic pollutants, and dyes. Bio-filtration and traditional activated sludge processes are less efficient in removing all types of contaminants [37]. Membrane treatment and Active Oxidation Processes (AOPs) have shown higher efficiency in removing organic pollutants from waters [38,39,40,41]. Compared with other treatment processes, adsorption technology has been widely utilized for its efficient and economic characteristics. Over the last few years, various practical and efficient adsorbents have been used for wastewater treatment [42,43,44]. In recent years, the research society has focused on improving limitations previously associated with the adsorption process, such as selectivity for removing pollutants [45,46]. 

According to Somma et al. [47], adsorption is an effective technique, and the most frequently used method regarding water quality and purification, as it offers high efficiency in both organic and inorganic pollutant removal, and has the potential to treat larger quantities of water for several purposes. The adsorption capacity is entirely dependent on such factors as contact time, concentration, dosage, kinetics, isotherm models and reaction conditions [48]. The porous structures of adsorbents also help in capturing pollutants from wastewater [49]. 

**Figure 1:**
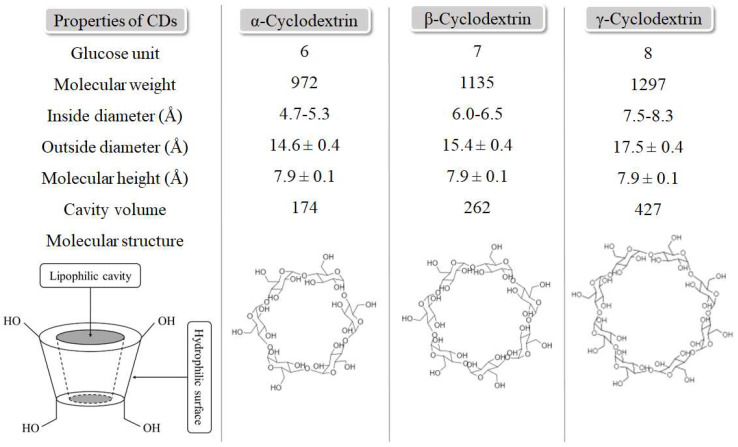
Properties and structures of cyclodextrins.

Some inexpensive and nontoxic polysaccharides from various sources have been moderately applied for environmental protection in recent years. Cyclodextrins (CDs) are cyclic polysaccharides and have been commonly used in textile, food, medicine, and other fields [50,51,52]. They were first synthesized by Antoine Villiers in the late 19th century [53], and the structures are shown in Figure 1. They were first used in the food and pharmaceutical industries and in chromatography in the 1980s, and then in other fields. In 1999, it was proved that CDs are non-toxic adsorbents by conducting research with the use of CD-containing materials [54]. Native CDs have primary and secondary hydroxyl groups in their structures. They can guarantee coordination sites that are able to chelate metal ions. In addition, OH groups at basic pH can be deprotonated and form covalent bonds [55]. Modified CD adsorbents provide novel structures for higher adsorption and address CD monomers’ limitations. In addition, novel CD adsorbents have been found effective for the simultaneous removal of several pollutants [56], and they were quickly separated while efficiently adsorbing [57].

This review paper aimed to overview various articles related to the adsorption of several pollutants by CD-based adsorbents and features the associated limitations and challenges. This paper summarizes developed synthetic methodologies and processes for the modification and preparation of CD adsorbents in detail. Moreover, CD-based adsorbents’ adsorption properties for particular pollutants, including heavy metal ions, pesticides, pharmaceutical residues, and dyes, are summarized. Furthermore, this paper explicitly addresses prospects, developments and challenges in applications.

## 2. Synthesis of Cyclodextrin-Based Adsorbents

**Figure 2:**
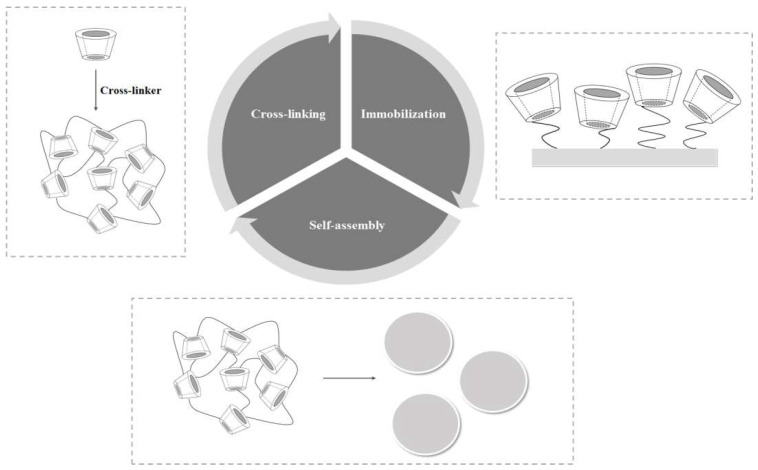
Synthesis scheme for CD-based materials.

CD is used as an additive for food and medicine application and is approved by the FDA [58,59]. CD is an oligosaccharide fabricate using enzymatic hydrolysis of starch, connected by α-1, 4-glycosidic bonds, which are divided into three types (α-, β-, and γ-CD) [60,61]. After producing enzymatic hydrolysis, experiments revealed that there are many other CD derivatives other than the three common types mentioned above, and their properties are also comparable to the most common CDs [62,63]. In addition, modification methods and synthesis can be split into three main classifications, and they are briefly discussed in the section below. The synthesis scheme is shown in Figure 2.

### 2.1. Cross-Linking

Cross-linking concerns reactions to get stable molecular structures where molecules are bonded to each other. The authors mainly discuss the cross-linkers for polymer adsorbents and their properties, along with the adsorption mechanism of several adsorbents. 

Cross-linking with epichlorohydrin has been widely used for over 50 years [64]. Another cross-linker, such as polyvinyl alcohol, was also considered for some practical applications, due to their low cost and easy preparation [65]. Chitosan (CS)-gamma CD, an adsorbent, has shown high stability and long life [66], proving to be a practical application. Similarly, CS-grafted beta-CD cross-linked using glutaraldehyde was confirmed to have characteristics and properties of CD and CS [67]. However, glutaraldehyde and EPI cross-linkers cause harm to the ecosystem and humans. They react with aromatic groups (rigid) and make them toxic. Various environmentally-friendly cross-linkers have gained much attention to remove contaminants. 

Ethylenediamine tetraacetic acid (EDTA) has been commonly used as a cross-linker. It is cross-linked with the adsorbents, and they have been effective for metal removal because of the chelating reaction between metals and EDTA [68]. Citric Acid (CA) has also become extensively utilized as a cross-linker in recent years. When CA cross-links adsorbents it results in majorly rich groups that are effective and higher in adsorption efficiency [69]. Conclusively, cyclodextrin-based adsorbents obtained using cross-linkers appear to be effective for removing metals, dyes, and other organic pollutants.

### 2.2. Immobilization

Immobilization involves immobilizing material on the solid supporter by physical or chemical methods. Besides the preparation concept, immobilization also refers to the different types of adsorbents with a macromolecular structure used as a supporter. Immobilization is dependent on the kind of supporter for CDs and is categorized into two separate parts: fiber composites and magnetic materials.

Fiber composites possess a stable property with several functional groups. CDs immobilized by the chemical cross-linking process primarily produce fibers. To prepare polymer fibers, N-isopropyl acrylamide (NIPAM) could settle cyclodextrin molecules to obtain a porous material, and it has been found to be helpful to remove more than a gram of crystal violet dye by a gram of adsorbent [70]. Moreover, electrostatic force has been a widely utilized method for immobilization. An environmentally-friendly composite fibrous adsorbent, synthesized from gelatin and β-CD molecules by the electrospinning method, has been used for Methylene Blue (MB) [71]. Carbon nanofibers have been used as adsorbents in several applications and have been used as carriers for decades. They have also shown a higher adsorption capacity when CD molecules are introduced. Furthermore, Li and co-authors prepared CD-based carbon nanoparticles via electrospinning. They gained a larger specific surface area, more porous structure, and larger pore volume than typical carbon nanofibers [72]. 

Natural fibers refer to materials derived from natural resources, like straw, cotton, and wood. CD was introduced onto cotton fibers by chemical interaction to enhance the adsorption capacity for Methyl Blue and Congo Red (CR) [73]. For dyes, macromolecules have good properties and have higher adsorption capacities when connected with β-CDs.

However, magnetic adsorbents have more practical applications, compared to fibrous composites. Iron oxide (Fe_3_O_4_), a standard magnetic material, has been extensively used as an excellent solid supporter [74] in the last decade. CD introduced with Fe_3_O_4_ has been extensively used to remove heavy metals and dyes. By chemical reaction, β-CD is modified on Fe_3_O_4_ NPs when reacted with CS (CDCM) [75]. Similarly, CD immobilized EPI cross-linked with Fe_3_O_4_ could be effective for dyes and heavy metals [76]. Therefore, graphene oxide (GO) has also been utilized as a magnetic support for the adsorbent, and this offered excellent removal of atrazine (ATZ) [77]. By using the magnetic properties of iron oxide, the adsorbent can be rapidly and easily extracted from water through magnetic attraction.

### 2.3. Self-Assembly

Self-assembly refers to the procedure of a system’s transition from an unorganized state to a well-structured state while the components are interacting, which happens in organisms and in nature [78,79]. CD-based molecules, when self-assembled, have been used for wastewater treatment because of the unique structure of the cavity of cyclodextrin. Self-assembled polymers were used to remove manifest pollutants, like imprinted molecules. Moreover, a β-CD-based adsorbent was synthesized into a film by adding bisphenol A (BPA) as a template molecule [80], and it showed excellent removal properties for BPA. In addition, CD molecules were embedded in the pore diameter of nano-porous carbon to acquire a self-assembly mechanism for CD functionalization and nano-porous carbon (ONC). Due to the availability of a specific cavity of CD molecules in the adsorbents, the pollutants were adsorbed quickly. However, it is challenging to use self-assembled adsorbents to treat more significant quantities. They have better efficiency for single pollutants, but are limited regarding multiple pollutant removal.

### 2.4. Characterization

The materials synthesized as described above were initially characterized by determining the content of the complex cyclodextrin, water absorption, point of zero charge, and decomposition temperature. The degree of cross-linking could be determined from the FTIR spectra. Optimum adsorption conditions were also determined, i.e., initial concentration of impurities and sorbents, contact time, pH, presence of salts and humic acids. Regeneration and characterization of the regenerated material were also carried out. All experimental points were adjusted to appropriate kinetic and adsorption models from which the main parameters, such as rate constants, equilibrium constants and maximum occupancy, were determined. Additionally, material samples could be subjected to other tests necessary for full material characterization, such as SEM, elemental analysis, BET isothermal analysis and thermogravimetric analysis. The final test is the use of real environmental samples.

## 3. The Mechanism and Function of CDs in Adsorbents

Cyclodextrin adsorbents are systems in which sugar macrocycles are part of the synthesized network. In this system, interactions with the adsorbate are based on several phenomena. The most important, explaining the legitimacy of using CD, is the inclusion of the guest molecule inside the torus [81,82,83]. Many organic molecules are present in environmental waters. As the interior of the CD is hydrophobic, matrix components that do not contain lipophilic elements are discriminated against. Most dyes and endocrine active molecules are aromatic compounds with at least one benzene ring that slips easily into the torus [84]. The outer part of the torus is hydrophilic and can participate in interactions involving functional groups formed during the reaction with the cross-linking agent [85]. If, for example, polyfunctional derivatives of hydroxy acids, amino acids or polyamines have been used in cross-linking, the adsorbent may additionally retain metals by chelation [86,87]. The selection of the cross-linking agent is crucial at the macromolecule design stage [88].

**Figure 3:**
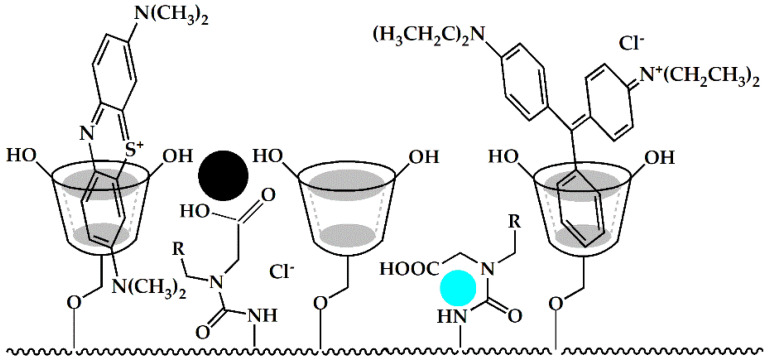
Fragment of the structure of the cyclodextrin adsorbent interacting with dyes and metal cations.

When the adsorption mechanism is based solely on the formation of supramolecular complexes, the amount of pollutant removed is the result of the number of available tori. Therefore, it is customary to specify the CD content of the material [89,90]. Moreover, the mechanism of cyclodextrin is shown in the Figure 3. It sometimes happens that the degree of capacity is significantly greater, which indicates the synergistic interaction of other elements of the structure [91]. The explanation may also be the formation of complexes with a different stoichiometry than assumed and based on research carried out with the use of native cyclodextrin solutions.

## 4. Application of Cyclodextrin-Based Adsorbents

### 4.1. Removal of Dyes

Dyes are being removed with high efficiency by using CD-based adsorbents. Various studies related to dye adsorption. Adsorption efficiency depends upon the interactions of analyte molecules with the adsorbent [92]. Various adsorbents have different abilities to uptake the dye in their matrix. The β-CD surface is rich in oxygen, and 0.1 m^2^/g surface area adsorbs CR and MB with a maximum capacity of 1.80 − 10^−2^ mmol/g but a higher affinity for CR, because of the CD and hydrophobic dye interactions. In contrast, MB has weak interaction of forces at the hydrophilic surface of β-CD [93].

**Figure 4:**
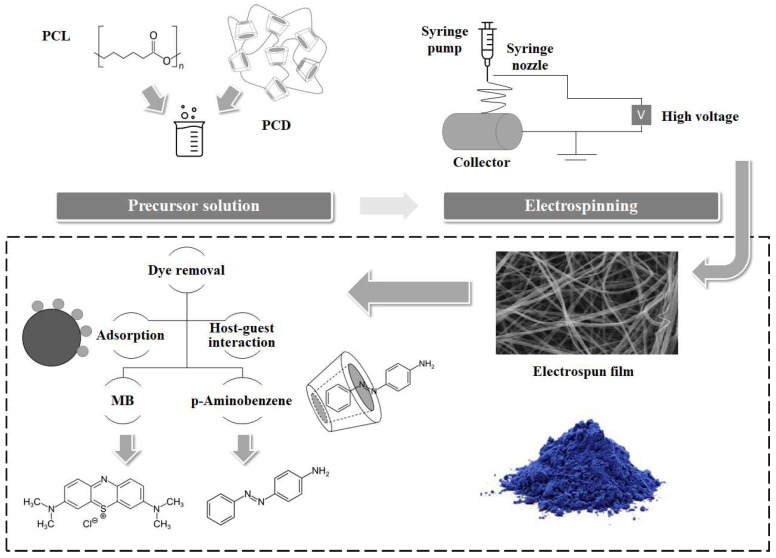
Schematic representation for the synthesis and adsorption mechanism of β-CD/PCL composite fibers for MB [95].

Host-guest complexation has become the advanced and economical adsorption technique. In a new study, β-CD and oil orange SS (OOSS) azo dye complexes were formed by the coprecipitation method to check their water purification ability. OOSS dye encapsulation with the β-CD hydrophobic cavity was confirmed by thermal analysis and FTIR. The complexation induced stability in the matrix, due to host-guest complex formation [94]. In another study, β-CD and ε-polycaprolactone (PCL) composite fibers were prepared by the electrospinning method through the host-guest complexation mechanism, as shown in Figure 4. The adsorbents were characterized by SEM, XRD, EDXS, and FT-IR. The synthesized fiber exhibited selective response for the 4-aminoazobenzene and MB solutions. The dye elusion efficiency was achieved at 24.1 mg/g towards 4-aminoazobenzene. Adsorption stability, sensitivity, and selectivity of the electropunk fibers encourage their potential use at a large scale for dye adsorption [95]. Natural polysaccharides can perform surface modification of GO due to various active functional groups. In a new study, the GO/β-CD composite was prepared by the cross-linking method to study its performance in MB adsorption. The new material was characterized by SEM, XRD, FT-IR, Raman and TGA analysis. GO/ β-CD exhibited the maximum adsorption capacity of 76.4 mg/g in six consecutive adsorption–desorption cycles [96].

**Figure 5:**
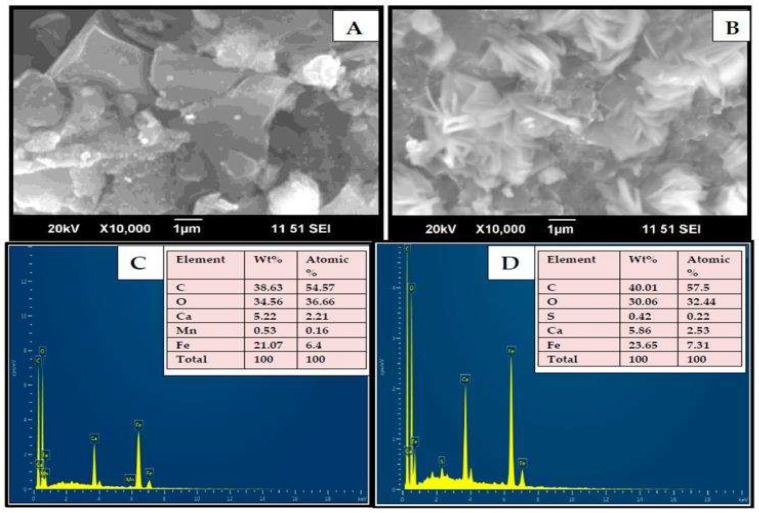
SEM-Energy Dispersive X-ray Spectrometry (EDX) images of Fe_3_O_4_/AC/CD/Alg polymer beads before (**A**) and after (**B**) adsorption. Fe_3_O_4_/AC/CD/Alg elemental analysis before (**C**) and after (**D**) adsorption [97].

Functionalized polymer composites gained much attention due to their high sensitivity, easy availability, good separation, and various active groups in the polymeric chain. Combining functionalized polymers with nanoparticles induces excellent properties in adsorbent materials. Yadav et al. reported a magnetic polymer nanocomposite by the addition of Fe_3_O_4_ nanoparticles (NPs) with β-CD/activated charcoal (AC)/sodium alginate (Alg). Figure 5 shows the SEM images and elemental analysis of the synthesized adsorbent. Langmuir isotherm fitted on the adsorption studies and prepared polymer composite exhibited the adsorption capacity of 10.63 mg g^−1^ for MB with an elution rate of 99.53% [97].

A combination of adsorption and membrane technology enhances the adsorbent performance. Li et al. modified filter paper with CD and citric acid to remove dual molecules, including dyes and Cu ions. Adsorption capacities of 39.1 mg g^−1^, 99.7 mg/g, 124.6 mg g^−1^, and 130.4 mg g^−1^ were determined for the Cu(II), Rhodamine-B (RB), Methylene Blue (MB), and Brilliant Green (BG), respectively. CD-based membranes can reduce the risk of various diseases if implanted in different dyes in affluent places and suggest new paths in the adsorption field, according to [98], which also discussed CD-based adsorbents’ interaction, complexation, and membrane mechanism for the removal of dyes. Every process has its positive aspects, and also the adsorption capacity depends upon some other factors like temperature and pH, depending on the adsorbate material composition. Furthermore, Table 1 summarizes the adsorption capacity and removal efficiencies of several CD-based adsorbents for dyes. 

### 4.2. Removal of Heavy Metals

**Figure 6:**
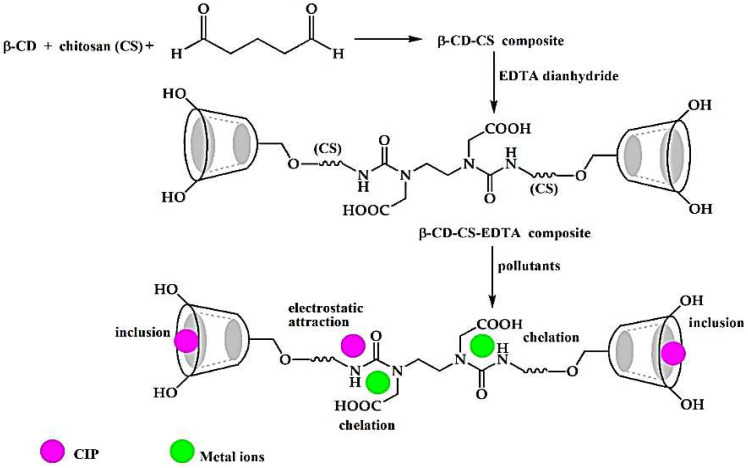
Schematic representation for the preparation of β-CD-Chitosan adsorbent [103].

Generally, metals can be eliminated through complexation, interaction, or ion exchange mechanisms. The efficiency of the adsorbent depends upon various factors, especially on the preparation technique and optimal pH. The CD polymer has deprotonated hydroxyl groups, which bind the metal strongly under primary conditions. The stability towards environmental conditions is enhanced by polymerizing Poly-CD/metal ion complex with polyacids. Anceschi reported Poly-CD/PVA fibers by the electrospinning of various Poly-CD and PVA solutions for the adsorption of Cu (II) ions. The fiber morphology was investigated by SEM, and physical-chemical properties were examined by FTIR and TGA. The ability of the insoluble CD-based fibers to eliminate heavy metals from wastewater was examined by analyzing the adsorption of Cu^2+^ and Cd^2+^ using ICP-OES. Poly-CD/PVA fibers exhibited an excellent response, especially towards the Cu(II), with a maximum adsorption capacity of 48.15 mg/g [101]. In another study, nano-sponges were fabricated through a complexation mechanism by cross-linking linecaps and CD with citric acid. Another pyromellitate nano-sponge was synthesized by the same reaction with pyromellitic dianhydride to compare the responses. The response was recorded at the 500 pm metal concentration, and pyromellitate exhibited a higher retention capability than the citrate-based nano-sponges. Pyromellitate adsorbent showed an adsorption capacity of 272 mg/g and 81 mg/g for the Pb(II) and Cu(II) [102]. Dual adsorbents are a new addition to adsorption technology. Single class pollutants have been reported, and it is challenging to eliminate organic and inorganic contaminants with the same adsorbents. Recently, Verma et al. reported β-CD-CS-EDTA adsorbent for the extraction of heavy metals (Ni(II), Cu(II), and Pb(II)) and ciprofloxacin (CIP). Figure 6 represents the synthesis mechanism of the adsorbent material. The adsorption mechanism was examined using EDX, FTIR, and elemental mapping methods. Heavy metals adsorption was more rapid than the CIP, with an adsorption capacity of 118.90, 161, and 330.90 mg/g for Ni(II), Cu(II), and Pb(II), respectively, while the CIP adsorption capacity was found to be 25.40 mg/g. It is a direction toward new research related to the elimination of organic and inorganic pollutants from water at the same time by a single adsorbent [103].

**Figure 7:**
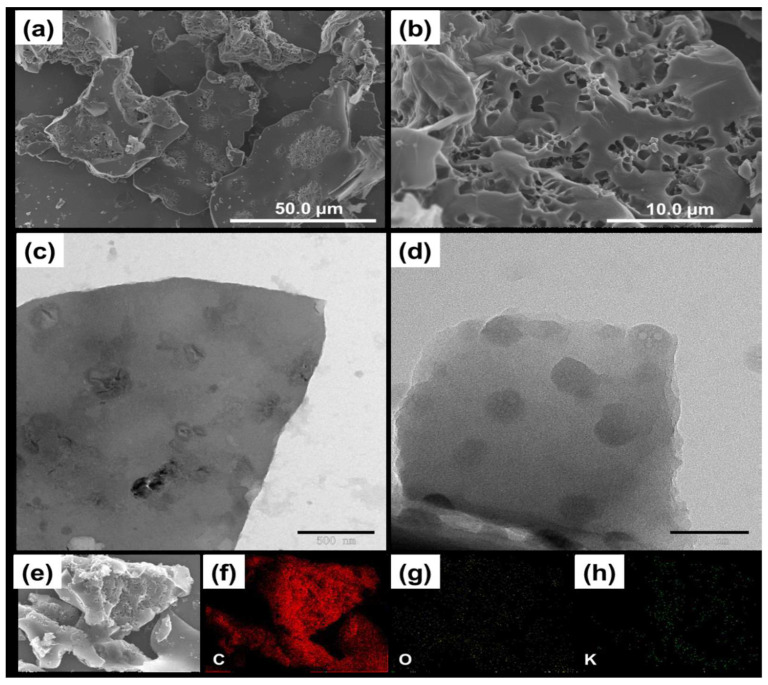
γ-CD MOF-NPC; SEM images (**a**,**b**), TEM images (**c**,**d**) and the mapping of C, K and O element(**e**–**h**) [109].

Magnetic composites are frequently used in adsorption due to their strong hydrophilicity, greater surface area, and various active sites [104,105]. The addition of magnetic composite with poly (vinylidene fluoride) PVDF membranes is due to the characteristics mentioned above. Zhang et al. modified PVDF with *β*-CD/GO/Fe_3_O_4_ to remove Cu(II) metal ions. The morphological and structural properties of the nanocomposite were investigated by TEM, XRD, FTIR, EDX and VSM techniques. The prepared adsorbent exhibited an adsorption capacity of 0.94 mg/g, and pH > 6 enhanced Cu’s elimination rate (II). The high-performance membranes, modified with biomaterial/nanoparticles composite, had excellent efficiency in adsorbing heavy metals from water [106]. Nano-porous carbons are getting research attention and are being frequently applied in adsorption applications, due to their various essential characteristics. Metal-organic framework (MOF) is a new class of nanoarchitecture particles with excellent stability and sensitivity [107,108]. Liu et al. prepared a g-CD-MOF composite by carbonizing the framework with potassium ions and γ-cyclodextrin. Figure 7 represents the SEM, TEM, and elemental mapping of the prepared adsorbent. The γ-CD-MOF-based adsorbent exhibited an adsorption capacity of 140.85 mg/g for Cd(II) ions that the Langmuir model calculated. The adsorption followed the ion exchange mechanism due to oxygen-comprising functional groups [109]. These studies revealed that CD has a remarkable ability to remove metal ions from water under laboratory conditions, making it a solid foundation for industrial research. Furthermore, Table 2 summarizes the adsorption capacity and removal efficiency for several heavy metals on CD-based adsorbents.

### 4.3. Removal of Organic Compounds

Along with the evolution of civilization, the progress of industrial processes is also taking place, due to the growing demands of people for resources, including resources for medicines and food. During the drug production process in pharmaceutical companies, an inseparable part of it is the generation of huge amounts of wastewater. Due to incomplete treatment, residues in sewage will be released into the environment and then into the human body, causing negative consequences over the years [116].

It is similar in the case of the food and packaging industry, which uses harmful endocrine-active compounds (EDCs) in the production of food packaging [117]. Due to increased temperature (e.g., when heating food), they enter the body along with food [118]. In addition, cans or plastic packages containing toxic EDCs often get into the water through inappropriate garbage handling. In the waters of the seas, lakes, and oceans, these toxic EDCs degrade into micro- and then nano-plastic, harming many species of animals, plants, and others. Therefore, endocrine-active compounds and drugs remain in, and accumulate in, polluted water, which has gradually attracted the attention of people [119]. Most organic pollutants in this section tend to bio-accumulate, persist in the waters for an extended time and are highly toxic to humans and animals [120]. Since cyclodextrins can self-adapt their structure to the structure of the adsorbed pollutant by reorganization, they are an ideal example of a material with great potential to remove organic contaminants from the environment. Adsorption of organic pollutants as a means of removal was tested on cyclodextrin-based materials [121]. Previous work has shown that cyclodextrins help remove endocrine-active compounds, pharmaceuticals, and other organic compounds [122,123]. Furthermore, removing the aforementioned groups of impurities involves model compounds, such as bisphenol A, nonylphenol, estrogen, carbamazepine, ibuprofen, naproxen and ciprofloxacin [124,125]. The maximum adsorption capacities and removal efficiencies of various CD-based adsorbents for selected organic compounds are listed in Table 3 and Table 4.

#### 4.3.1. Endocrine Disruptors

Bisphenol A (BPA) is a commercial substance from a group of phenols that have been commonly used since the 1960s. For years, BPA has been used in the chemical industry, particularly in producing polycarbonate plastics and resins [126,127]. Exposure to bisphenol A may affect the occurrence of, among other things, diabetes, obesity, metabolic syndrome, cancer, and fertility disorders [128]. To remove chemicals that disrupt the functioning of the endocrine system, CD-MG, mesoporous magnetic clusters connected with β-cyclodextrin, were synthesized and studied. Adsorption isotherms showed that BPA adsorption was 52.7 mg/g. In addition, the adsorbent showed an admirable quality of 84.5% in reusability test experiments after four cycles. Their quick process kinetics, delightful adsorption capacity, and CD-MG adsorption mechanism result in high potential for removing active endocrine compounds from the environment [120].

Another work presented an ecological and straightforward method of producing porous poly (chloromethyl styrene) resin modified with β-cyclodextrin with a spongy cross-linked material, namely PS@CM-CDP, for the ultrafast and effective removal of organic contamination from aquatic solutions. The obtained PS@CM-CDP, which stood out as having maximum adsorption capacity for BPA, could reach beyond 8.25 mg/g. In addition, the circular identity of the resin rendered native β-CD polymers reusable, even after six usages, without significant performance degradation. The paper also presented the practical application of the obtained material, which illustrated multiple perspectives on the water treatment column [129].

In work from 2021, a cyclodextrin material was designed, produced, and used to eliminate Pb (II) and BPA, which may coexist in the aquatic environment. The characterization outcomes corroborated successful β-CD grafting and Fe_3_O_4_ loading. Moreover, the resulting material β-CD@MRHC had commendable magnetic properties for efficient regeneration from water, unaffected by pollutant adsorption. The synthesized material showed excellent adsorption efficiency with a maximum bisphenol A uptake of 412.8 mg/g, and it only took 7.5 min to achieve adsorption equilibrium. In addition, the material accomplished synergistic elimination of Pb(II) and BPA by reversing their competing behaviors, thanks to different holding mechanisms. The adsorbent was found to be promising and suitable for practical use in the simultaneous elimination of heavy metals and organic matter from water resources, employing high-efficiency magnetic recovery [130].

Moreover, Bucur S. et al. synthesized silica particles with β-cyclodextrin that showed the ability to remove bisphenol A (BPA) from sewage water. It turned out that this was related to the presence of β-CD groups on the silica surface. The experiments showed that the qe values were 107 mg/g for SiO_2_-β-CD-OH and 112 mg/g for SiO_2_-β-CD-NH_2_, respectively, while adsorption equilibrium was reached after 180 min [116]. The bagasse-β-cyclodextrin polymer, namely SB-β-CD, accounted for 121 mg/g [131], and showed similar values of the maximum BPA adsorption capacity.

**Table 3:** Maximum adsorption capacities and removal efficiencies of various CD-based adsorbents for selected endocrine disrupting compounds.

Adsorbent	Pollutant	q_max_	Removal Efficiency [%]	References
β-CDPs	Bisphenol A	460.0	>99.9	[126]
GO-β-CD nanocomposites	Bisphenol A	373.4	95.0	[127]
Hyper-cross-linked β-CD porous polymer	Bisphenol A	278.0	78.8	[128]
Zr/CM-β-CD	Estrogens estradiol	210.5	>97.0	[112]
β-CD-TFP	Bisphenol A	164.4	98.1	[129]
β-CD-DFDS	Bisphenol A	113.0	90.0	[130]
Diatomite cross-linked BCD polymers	Bisphenol A	83.6	91.2	[131]
β-CD polymer-functionalized Fe_3_O_4_ magnetic nanoparticles	Bisphenol A	74.6	41.0	[132]
β-CD-Functionalized Mesoporous Magnetic Clusters	Bisphenol A	52.7	84.5	[133]
CS-ED-CD	Bisphenol S	44.3	87.0	
β-CD-modified graphene oxide membranes	Bisphenol A	25.5	100	[134]
β-CD-poly(glycidyl methacrylate)-SiO_2_-nanoparticles	Bisphenol A	22.5	97.2	[135]
β-CD-alginate	Nonylphenol	22.0	46.0	[136]
GPP	Dialkyl phthalates	6.6	72.9	[137]

Synthetic zeolite with NaX structure was modified with β-cyclodextrin (CD) and used for adsorption of, for example, BPA and ibuprofen from model solutions. The best fit to the Langmuir model shown in adsorption experiments indicated monolayer adsorption and the formation of inclusion complexes with hydrogen bonding. The researchers mentioned that the qmax parameter was 32.7 mg/g for BPA. The adsorptive behavior of NaX-CD designated that it could be a skillful adsorbent for the eradication of EDC pollutants from contaminated water [132].

A new β-CD modified graphene oxide membrane (CDGO) was synthesized in 2019 to effectively remove bisphenol-A. The CDGO membranes were produced by vacuum filtration and laying CDGO nanosheets onto spongy compounds. Nanosheets were produced by chemically grafting β-CD particles on both sides of graphene oxide nanosheets. The BPA adsorption efficiency by the synthesized material wss several times higher than that of the affinity membranes that were earlier used. In addition, this material was readily regenerated by treatment with ethanol. After a few cycles, it was possible to restore the effectiveness of BPA removal to almost 100% [123].

In 2020, a porous membrane made of cellulose nanofibers with modified β-cyclodextrin (CA-P-CDP) was produced and tested. The impurities used were model bisphenol compounds bisphenol A, bisphenol S, and bisphenol F. In addition, temperature, adsorbent dosage, and pH were tested during the adsorption process, and it was shown that they had a significant effect on the adsorption efficiency. Under chosen conditions (25 °C, 7.0 pH, and 0.1 g/L dose of material), the removal of model pollutants reached equilibrium within 15 min, and the qmax parameter was 50.37, 48.52, and 47.25 mg/g for bisphenol A, S and bisphenol F, respectively. The work focused on the interaction of hydrophobic effects, hydrogen bond interactions, and π-π stacking interactions. In addition, it was proved that the amount of treated solution by CA-P-CDP was 14.5 times greater than that of a pristine-cellulose membrane. In addition, the authors also indicated the complete removal of pollutants during the treatment of natural water samples, which proved that the tested material could be used in practice [133].

Another example of the application of CD-based adsorbents may be using a new, environmentally friendly adsorbent formed by cross-linking with β-cyclodextrin diatomite (DA@β-CD) for the removal of EDCs. The synthesized DA@β-CD had 1.4 to 2.5 times higher BPA removal capacity than DA. The test material was steady and reusable after washing with an alkaline solution. A removal held after three regeneration cycles, still achieved a stable removal rate of 91.15% [118].

Cyclodextrin-based materials can remove many contaminants other than BPA. For example, in one study, Fe_3_O_4_@CD MNPs were produced by chemical co-precipitation, and could quickly be isolated from the water phase by a magnet. The efficiency of 1-naphthol removal by the mentioned material was tested according to the batch technique. The qmax parameter of Fe_3_O_4_@CD MNPs was higher than that of several adsorbing materials tried before. Moreover, the paper showed the cost-effective and advantageous removal of Co(II) and 1-naphthol simultaneously by Fe_3_O_4_@CD MNPs [134].

In addition, core-shell nanoparticles can also be used to adsorb harmful compounds. An example may be the magnetic and photocatalytic Fe_3_O_4_@TiO_2_ prepared and described by researchers. It is formed when cyclodextrin cavities are anchored in TiO_2_ coating. The model pollutant used in the study was butyl phthalate. An undeniable advantage of this type of material was the possibility of separating the functionalized nanoparticles by magnetic action and reusing them. The dispersibility of nanoparticles in water captured organic impurities, due to the presence of cyclodextrin in the composite material. Moreover, the desirable feature of these adsorbents was that they could be removed entirely and reused, while maintaining the current adsorption capacity [135].

Researchers also undertook the removal of estrogens (including estradiol and estrone) from the environment using Zr/CM-βCD, i.e., the Zr(IV)-cross-linked carboxymethyl-β-cyclodextrin, which was constructed by an easy, all-purpose and environmentally friendly method. Namely, this material could be obtained by a chelation reaction between Zr(IV) and the carboxyl group of the cyclodextrin used. Furthermore, the CD cavities encapsulated estrogenic contaminants through inclusion complexes. It was also possible to remove metals simultaneously with this material as it has been suggested that the carboxyl groups should react by chelating metal ions. In the one-component system, the qmax parameter of Zr/CM-β-CD towards estradiol was 210.53 mg/g. The adsorption capacity of the Zr/CM-β-CD remained high after five cycles. Interestingly, the material was very economical at concurrent removal of EDCs and metals [98].

The mechanism of EDC adsorption by CD-based adsorbents is different than that for the removal of heavy metals and dyes described in this paper. The CD cavity does not have the role of enriching the uptake of contaminants but also plays a role as the location of in situ catalytic degradation [136]. On the other hand, selective matching between guest and host can be a limiting step in the adsorption process [137].

#### 4.3.2. Pharmaceutical

Pharmaceutical contaminants have been found in water bodies and pollute the entire ecosystem. For example, carbamazepine, a dibenzoazepine derivative, is used as a psychotropic, anticonvulsant, and mood-stabilizing drug, mainly to treat epilepsy and bipolar disorder. The drug is widely detected in European and North American water [138]. Its presence has also been demonstrated in green algae, crustaceans, stinging beetles, and hydroids [139]. This toxic compound is tough to degrade [140]. For this reason, studies have been conducted that showed that the maximum adsorption capacity (q_max_) of carbamazepine by cyclodextrin material is 136.4 mg/g [141].

The above-described case of finding pharmaceuticals in water is not an isolated one. Another example is non-steroidal anti-inflammatory drugs (NSAIDs), which include ibuprofen, ketoprofen, and diclofenac. In one of the articles, the authors used CDs combined with nano-filters to eliminate these pollutants from the aquatic environment. The tested nano-filters were of different thicknesses and chemical compositions. In the study, the maximum adsorption capacity was estimated using ibuprofen as a model by measuring the removal of pharmaceutical residues from municipal wastewater. After use, nano-filters can be regenerated using ethanol, and the q_max_ of chemicals can be improved [142]. A notable example of attempts to remove NSAIDs from water is the use of CD polymer as the third step in treating biomechanical municipal wastewater in a wastewater treatment plant. Fenyvesi and colleagues found a very high level of contamination removal in a relatively short time [143].

In another study, the hydrothermal method was used to obtain a composite of reduced graphene oxide immobilized with β-cyclodextrin (β-CD/rGO) in one step to eliminate naproxen from water. The material with a high porosity structure with numerous hydroxyl groups showed the stable removal of NSAID and an excellent adsorption capacity of 361.85 mg/g at 313 K temperature [144].

In another research, innovative Fe_3_O_4_/CD/AC/SA polymer gel beads, consisting of functionalized iron oxide activated carbon particles, sodium alginate polymer, and β-cyclodextrin, were obtained by a straight, repeatable, and cheap method. The maximum adsorption capacity of this material was 3.125 mg/g for ciprofloxacin, the most potent drug among the fluoroquinolones, with a bactericidal effect, showing its activity by inhibiting bacterial DNA topoisomerase and DNA gyrase [124]. Moreover, Fe_3_O_4_/CD/AC/SA can be regenerated and smoothly separated from solutions without losing removal efficiency. After regeneration, they have the same adsorption capacity for up to four desorption and adsorption cycles [145]. The problem of the presence of drugs in sewage can be reduced by absorbing the substances with derivatives of cyclodextrin polymers. The CD polymer/pulsed light sequential method was tested for contaminant removal to overcome this problem, and the simulations showed that the cyclodextrin 3D network gives the CD polymer amphiphilic features, which help to reduce the concentration of impurities by 77%. Moreover, after using the CD polymer on contaminated water, the authors treated it with pulsating light, by which the presence of contaminants was further reduced by up to 91%. The CD polymer was shown to be reusable at least ten times while eliminating one of the most popular NSAIDs, ibuprofen. For this reason, a CD polymer system, combined with pulsating light, can be an innovative and efficient method of removing NSAIDs from water [146].

**Table 4:** Maximum adsorption capacities and removal efficiencies of various CD-based adsorbents for selected pharmaceutical compounds.

Adsorbent	Pollutant	q_max_	Removal Efficiency [%]	References
CNPs	Tetracycline (TC)	543.5	93.8	[72]
β−CD−M	Ibuprofen	86.2	90.0	[147]
CS-ED-CD	Procaine	48.0	86.0	[148]
	Imipramine	47.1	85.0	
	Ciprofloxacin	47.1	83.0	
β-CD/rGO	Naproxen	361.9	79.3	[144]
β-CD COF	Naproxen	-	98.0	[149]
	Ibuprofen	-	78.0	
Ca(II)-doped chitosan/β-CD composite	Acetaminophen	200.9	99.9	[150]
CD@Clay-PVP	Ibuprofen	3.46	83.9	[151]
β-CD polymer with tetrafluoroterephthalonitrile	Carbamazepine	136.4	65.0	[83]
GD-EDTS	Salbutamol	140.2	71.3	[146]
	Atenolol	236.9	70.4	

Drugs often used to treat chronic diseases, such as Salbutamol (SAL) and Atenolol (ATL), were tested in 2021 for their removal from the environment. Unfortunately, traditional wastewater treatment plants cannot cope with their complete removal. To efficiently remove the above-mentioned compounds, silanized adsorbents of β-cyclodextrin compounds (GD-EDTS) were designed and tested. Due to the introduction of carboxyl groups into this composite, it was possible to sustain satisfactory adsorption efficiency over a broad pH range. The maximum SAL and ATL adsorption capacities were 140.24 mg/g and 236.92 mg/g. Moreover, the poor rivalries behavior between SAL and ATL resulted in the fact that both impurities could be removed simultaneously with the test adsorbent.

## 5. Regeneration

**Figure 8:**
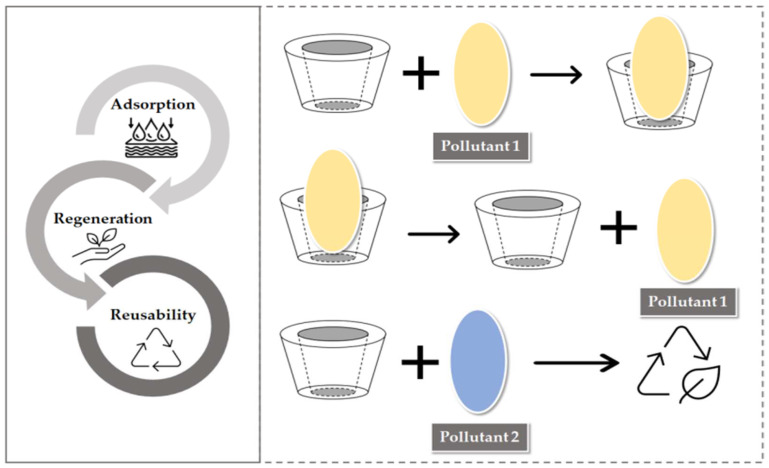
Schematic diagram of adsorption-regeneration-reusability.

Regeneration of an adsorbent plays a vital part in the determination of its efficiency and longer usage. Many adsorbents have been prepared but due to their lacking regeneration properties, they were not popular among researchers. Regeneration is always a key challenge (Figure 8). In most studies, solvent regeneration has been used for pollutants [152]. However, the efficiency of regeneration depends on the pollutant’s solubility in the solvent for regeneration [153,154]. In addition, to regenerate the adsorbent’s activity, the solvent regeneration process is used in β-CD functionalized adsorbents, However, it has been reported in many studies that the adsorbents lose their capacity to adsorb after each cycle [154,155]. In the adsorption/desorption part of a study by Qiang Lin and co-authors, in 2019, it was revealed that B-CD grafted cellulose beads were regenerated easily in methanol [82]. In another research by Usman and co-authors, in 2021, regeneration revealed that NTA-B-CD-CS acquired remarkable potential to treat and model wastewater several times [87]. Furthermore, it was reported in research that P-CDEC has shown no such decrease in removal efficiency for Ni(II) and BPA when compared to other polymers, and its regeneration was more than 90%, even after the fifth cycle [86].

## 6. Conclusions

Environmental pollution induces toxicity and affects human health. Researchers are exploring new routes to resolve issues of environmental pollution to regain the safe environment and ecosystem that existed before the commencement of pollution. Most pollutants, such as heavy metals, organic contaminants, and others, are bio-accumulated in food chains through water sources and ultimately disturb natural water bodies. These pollutants are resistant to degradation and accumulate in the biological system to produce toxicity, side effects, and diseases. Researchers have developed various technologies to reduce contaminants in the environment, and to protect the environment. Moreover, adsorption has gained considerable attention around the globe due to its unique characteristics. Macrocyclic molecules adsorb pollutants and ease multiple environmental and health concerns. CD is frequently used for adsorption due to its critical feature of water solubility for water remediation. Various CD-based adsorbents have been summarized in this paper, indicating recent progress in adsorption technology and the identification of research gaps to make the environment safe and healthy. The structural features of three types of cyclodextrins have been described, and finally an overview of the adsorption of heavy metals, dyes, pharmaceuticals and other organic contaminants by CD was provided. In addition, the modification of CD with nanoparticles, membranes, and fibers enhances the adsorption capacity and efficiency of the adsorbent material. CD adsorbents prepared by cross-linking have larger surface area and also a three-dimensional porous structure that is beneficial for the treatment of wastewater. Furthermore, hydrogen bonding, host-guest networks, electrostatic forces, and hydrophobic and acid-base interactions between CD and pollutants make the adsorption mechanism efficient. Many future aspects need to be explored for the commercial use of CD-based adsorbents. Although CD possesses several advantages over many adsorbents, many problems are still associated with CD composites. CD is a natural substance, but the green composites products. and their sustainability, remain a challenge for researchers, and there is a dire need for further studies. Degradation and toxicological studies need to be explored to make CD a versatile and commercial adsorbent. Adsorption on real water samples is also a key challenge because experiments are still performed at the laboratory level, so, there is a lot of work remaining to make CD-based adsorbents at an industrial level. Conclusively, any concerns related to CD-based adsorbents, in terms of low-cost, eco-friendliness, practicality, reproducibility, long-term stability, and reusability should be resolved for application at industrial levels.

## Figures and Tables

**Table 1:** Adsorption capacities of various CD-based adsorbents for dyes.

Adsorbent	Dye	Adsorption Capacity	Removal Efficiency (%)	pH	References
Fe3O4/AC/CD/Alg	MB	10.63 mg/g	99.53	6.0	[97]
β-CD/GO	MB	76.4 mg/g	90.00	7.0	[96]
β-CD filter paper	MB	124.6 mg/g	92.94	5.5–6	[98]
	BG	130.4 mg/g,		5.5–6	
	RB	99.7 mg/g		5.5–6	
Fe3O4/b-CD/GO	MG	990.1	98.00	7.0	[99]
Fe3O4-PEI/b-CD	MO	192.2	83.90	1	[76]
β-CD	CR	12 mg/g	65.00	-	[93]
PA-β-CD	MB	1095	90.00	6–10	[100]
PA-β-CD	BG 4	2005.58		6–10	
	CV	1930.23		6–10	

**Table 2:** Adsorption capacities of various CD-based adsorbents for metals.

Adsorbent	Dye	Adsorption Capacity (mg/g)	Removal Efficiency (%)	pH	References
Poly-CD/PVA	Cu(II)	48.15	85.00	8	[101]
β –PMDA	Pb(II)	272	95.00	-	[102]
β-CD-CS-EDTA	Pb(II)	330.90	-	5	[103]
	Cu(II)	161		5	
	Ni(II)	118.9	95.00	5	
Fe_3_O_4_/ GO (*β*-MGO)	Cu(II)	0.94 mg/g	93.14	6–7	[110]
α-CD MOF-NPC	Cd(II)	140.85	90.00	4	[109]
β -CDs polymer	Pb(II)	196.4	83.30	2	[111]
Zr/CM- β-CD	Cd(II)	118.3	-	7.0	[112]
Fe_3_O_4_-PEI/ β-CD	Pb(II)	73.1	73.40	6.0	[113]
CD-Fe_3_S_4_	Pb(II)	256.0	74.47	6.0	[114]
β –CDPP	Pb(II)	576.92	95.00	2	[115]

## Data Availability

Not applicable.

## References

[1] Gleick P.H. (2009). Basic Water Requirements for Human Activities: Meeting Basic Needs. Water Int..

[2] Qadir M., Sharma B.R., Bruggeman A., Choukr-Allah R., Karajeh F. (2007). Non-Conventional Water Resources and Opportunities for Water Augmentation to Achieve Food Security in Water Scarce Countries. Agric. Water Manag..

[3] Ghaffar A., Zhang L., Zhu X., Chen B. (2018). Porous PVdF/GO Nanofibrous Membranes for Selective Separation and Recycling of Charged Organic Dyes from Water. Environ. Sci. Technol..

[4] Chen M., Xu P., Zeng G., Yang C., Huang D., Zhang J. (2015). Bioremediation of Soils Contaminated with Polycyclic Aromatic Hydrocarbons, Petroleum, Pesticides, Chlorophenols and Heavy Metals by Composting: Applications, Microbes and Future Research Needs. Biotechnol. Adv..

[5] Demirbas A. (2008). Heavy Metal Adsorption onto Agro-Based Waste Materials: A Review. J. Hazard. Mater..

[6] Wang W., Gao H., Jin S., Li R., Na G. (2019). The Ecotoxicological Effects of Microplastics on Aquatic Food Web, from Primary Producer to Human: A Review. Ecotoxicol. Environ. Saf..

[7] Author D.T., Gisela S. (2011). Enhanced Wastewater Treatment by Ozone and Ferrate Kinetics, Transformation Products and Full-Scale Ozonation.

[8] Mulbry W., Kondrad S., Pizarro C., Kebede-Westhead E. (2008). Treatment of Dairy Manure Effluent Using Freshwater Algae: Algal Productivity and Recovery of Manure Nutrients Using Pilot-Scale Algal Turf Scrubbers. Bioresour. Technol..

[9] Mouele E.S.M., Tijani J.O., Fatoba O.O., Petrik L.F. (2015). Degradation of Organic Pollutants and Microorganisms from Wastewater Using Different Dielectric Barrier Discharge Configurations—A Critical Review. Environ. Sci. Pollut. Res..

[10] Maletz S., Floehr T., Beier S., Klümper C., Brouwer A., Behnisch P., Higley E., Giesy J.P., Hecker M., Gebhardt W. (2013). In Vitro Characterization of the Effectiveness of Enhanced Sewage Treatment Processes to Eliminate Endocrine Activity of Hospital Effluents. Water Res..

[11] Kasprzyk-Hordern B., Dinsdale R.M., Guwy A.J. (2009). The Removal of Pharmaceuticals, Personal Care Products, Endocrine Disruptors and Illicit Drugs during Wastewater Treatment and Its Impact on the Quality of Receiving Waters. Water Res..

[12] Gültekin I., Ince N.H. (2007). Synthetic Endocrine Disruptors in the Environment and Water Remediation by Advanced Oxidation Processes. J. Environ. Manag..

[13] Frisbie S.H., Mitchell E.J., Dustin H., Maynard D.M., Sarkar B. (2012). World Health Organization Discontinues Its Drinking-Water Guideline for Manganese. Environ. Health Perspect..

[14] Rasheed T., Bilal M., Nabeel F., Adeel M., Iqbal H.M.N. (2019). Environmentally-Related Contaminants of High Concern: Potential Sources and Analytical Modalities for Detection, Quantification, and Treatment. Environ. Int..

[15] Gupta R.C. (2020). Handbook of Toxicology of Chemical Warfare Agents.

[16] Sacco O., Vaiano V. (2019). Visible Light Active Structured Photocatalysts for the Removal of Emerging Contaminants: Science and Engineering.

[17] Kılıç O., Boz İ., Eryılmaz G.A. (2020). Comparison of Conventional and Good Agricultural Practices Farms: A Socio-Economic and Technical Perspective. J. Clean. Prod..

[18] Stuart M., Lapworth D., Crane E., Hart A. (2012). Review of Risk from Potential Emerging Contaminants in UK Groundwater. Sci. Total Environ..

[19] Vela N., Fenoll J., Garrido I., Pérez-Lucas G., Flores P., Hellín P., Navarro S. (2019). Reclamation of Agro-Wastewater Polluted with Pesticide Residues Using Sunlight Activated Persulfate for Agricultural Reuse. Sci. Total Environ..

[20] Marican A., Durán-Lara E.F. (2018). A Review on Pesticide Removal through Different Processes. Environ. Sci. Pollut. Res. Int..

[21] Saleh I.A., Zouari N., Al-Ghouti M.A. (2020). Removal of Pesticides from Water and Wastewater: Chemical, Physical and Biological Treatment Approaches. Environ. Technol. Innov..

[22] Ahmed M.B., Zhou J.L., Ngo H.H., Guo W., Thomaidis N.S., Xu J. (2017). Progress in the Biological and Chemical Treatment Technologies for Emerging Contaminant Removal from Wastewater: A Critical Review. J. Hazard. Mater..

[23] Lu J., Wang T., Zhou Y., Cui C., Ao Z., Zhou Y. (2019). Dramatic Enhancement Effects of L-Cysteine on the Degradation of Sulfadiazine in Fe^3+^/CaO_2_ System. J. Hazard. Mater..

[24] Lee H.S., Tang Y., Rittmann B.E., Zhao H.P. (2018). Anaerobic Oxidation of Methane Coupled to Denitrification: Fundamentals, Challenges, and Potential. Crit. Rev. Environ. Sci. Technol..

[25] Abbas A., Al-Amer A.M., Laoui T., Al-Marri M.J., Nasser M.S., Khraisheh M., Atieh M.A. (2016). Heavy metal removal from aqueous solution by advanced carbon nanotubes: Critical review of adsorption applications. Sep. Purif. Technol..

[26] Jiang Y., Liu B., Xu J., Pan K., Hou H., Hu J., Yang J. (2017). Cross-Linked Chitosan/β-Cyclodextrin Composite for Selective Removal of Methyl Orange: Adsorption Performance and Mechanism. Carbohydr. Polym..

[27] Yu Y., Yu L., Koh K.Y., Wang C., Chen J.P. (2018). Rare-Earth Metal Based Adsorbents for Effective Removal of Arsenic from Water: A Critical Review. Crit. Rev. Environ. Sci. Technol..

[28] Zou Y., Wang X., Khan A., Wang P., Liu Y., Alsaedi A., Hayat T., Wang X. (2016). Environmental Remediation and Application of Nanoscale Zero-Valent Iron and Its Composites for the Removal of Heavy Metal Ions: A Review. Environ. Sci. Technol..

[29] O’Connor D., Hou D., Ok Y.S., Song Y., Sarmah A.K., Li X., Tack F.M.G. (2018). Sustainable in Situ Remediation of Recalcitrant Organic Pollutants in Groundwater with Controlled Release Materials: A Review. J. Control Release.

[30] Peng W., Li H., Liu Y., Song S. (2017). A Review on Heavy Metal Ions Adsorption from Water by Graphene Oxide and Its Composites. J. Mol. Liq..

[31] Zhao G., Huang X., Tang Z., Huang Q., Niu F., Wang X. (2018). Polymer-Based Nanocomposites for Heavy Metal Ions Removal from Aqueous Solution: A Review. Polym. Chem..

[32] Juned M., Ahmed K., Ahmaruzzaman M. (2016). A Review on Potential Usage of Industrial Waste Materials for Binding Heavy Metal Ions from Aqueous Solutions. J. Water Process Eng..

[33] Priyadarshini E., Pradhan N. (2017). Gold Nanoparticles as Efficient Sensors in Colorimetric Detection of Toxic Metal Ions: A Review. Sens. Actuators B Chem..

[34] Bansod B.K., Kumar T., Thakur R., Rana S., Singh I. (2017). A Review on Various Electrochemical Techniques for Heavy Metal Ions Detection with Different Sensing Platforms. Biosens. Bioelectron..

[35] Vezzone M., Cesar R., de Souza Abessa D.M., Serrano A., Lourenço R., Castilhos Z., Rodrigues A.P., Perina F.C., Polivanov H. (2019). Metal Pollution in Surface Sediments from Rodrigo de Freitas Lagoon (Rio de Janeiro, Brazil): Toxic Effects on Marine Organisms. Environ. Pollut..

[36] Gao F., Li J., Sun C., Zhang L., Jiang F., Cao W., Zheng L. (2019). Study on the Capability and Characteristics of Heavy Metals Enriched on Microplastics in Marine Environment. Mar. Pollut. Bull..

[37] Tran H.N., You S.J., Hosseini-Bandegharaei A., Chao H.P. (2017). Mistakes and Inconsistencies Regarding Adsorption of Contaminants from Aqueous Solutions: A Critical Review. Water Res..

[38] Buruga K., Song H., Shang J., Bolan N., Jagannathan T.K., Kim K.H. (2019). A Review on Functional Polymer-Clay Based Nanocomposite Membranes for Treatment of Water. J. Hazard. Mater..

[39] Ismail M., Wu Z., Zhang L., Ma J., Jia Y., Hu Y., Wang Y. (2019). High-Efficient Synergy of Piezocatalysis and Photocatalysis in Bismuth Oxychloride Nanomaterial for Dye Decomposition. Chemosphere.

[40] Zhou Y., Zhang R., Chen K., Zhao X., Gu X., Lu J. (2017). Enhanced Adsorption and Photo-Degradation of Bisphenol A by β-Cyclodextrin Modified Pine Sawdust in an Aquatic Environment. J. Taiwan Inst. Chem. Eng..

[41] Zhou Y., Fang X., Wang T., Hu Y., Lu J. (2017). Chelating Agents Enhanced CaO_2_ Oxidation of Bisphenol A Catalyzed by Fe^3+^ and Reuse of Ferric Sludge as a Source of Catalyst. Chem. Eng. J..

[42] Zhou Y., Gu X., Zhang R., Lu J. (2014). Removal of Aniline from Aqueous Solution Using Pine Sawdust Modified with Citric Acid and β-Cyclodextrin. Ind. Eng. Chem. Res..

[43] Zhou Y., Gu X., Zhang R., Lu J. (2015). Influences of Various Cyclodextrins on the Photodegradation of Phenol and Bisphenol A under UV Light. Ind. Eng. Chem. Res..

[44] Zhou Y., Zhang R., Gu X., Zhao Q., Lu J. (2015). Sorption Characteristics of Phenanthrene and Pyrene to Surfactant-Modified Peat from Aqueous Solution: The Contribution of Partition and Adsorption. Water Sci. Technol..

[45] Chang Y., Shen C., Li P.Y., Fang L., Tong Z.Z., Min M., Xiong C.H. (2017). Optimization of Polyacrylonitrile–Cysteine Resin Synthesis and Its Selective Removal of Cu(II) in Aqueous Solutions. Chin. Chem. Lett..

[46] He W., Li N., Wang X., Hu T., Bu X. (2018). A Cationic Metal-Organic Framework Based on {Zn4} Cluster for Rapid and Selective Adsorption of Dyes. Chin. Chem. Lett..

[47] Somma S., Reverchon E., Baldino L. (2021). Water Purification of Classical and Emerging Organic Pollutants: An Extensive Review. ChemEngineering.

[48] Iravani S., Varma R.S. (2022). Nanosponges for Water Treatment: Progress and Challenges. Appl. Sci..

[49] Jia J., Wang C., Li Y., Wu D., Yu J., Gao T., Li F. (2022). Water-Insoluble Cyclodextrin-Based Nanocubes for Highly Efficient Adsorption toward Diverse Organic and Inorganic Pollutants. Sep. Purif. Technol..

[50] Uekama K., Hirayama F., Irie T. (1998). Cyclodextrin Drug Carrier Systems. Chem. Rev..

[51] Tian B., Hua S., Liu J. (2020). Cyclodextrin-Based Delivery Systems for Chemotherapeutic Anticancer Drugs: A Review. Carbohydr. Polym..

[52] Chen X., Chen M., Xu C., Yam K.L. (2019). Critical Review of Controlled Release Packaging to Improve Food Safety and Quality. Crit. Rev. Food Sci. Nutr..

[53] Liu Q., Zhou Y., Lu J., Zhou Y. (2020). Novel cyclodextrin-based adsorbents for removing pollutants from wastewater: A critical review. Chemosphere.

[54] Ko S.O., Schlautman M.A., Carraway E.R. (1999). Partitioning of Hydrophobic Organic Compounds to Hydroxypropyl-β-Cyclodextrin: Experimental Studies and Model Predictions for Surfactant-Enhanced Remediation Applications. Environ. Sci. Technol..

[55] Prochowicz D., Kornowicz A., Justyniak I., Lewiński J. (2016). Metal Complexes Based on Native Cyclodextrins: Synthesis and Structural Diversity. Coord. Chem. Rev..

[56] Qin X., Bai L., Tan Y., Li L., Song F., Wang Y. (2019). β-Cyclodextrin-Crosslinked Polymeric Adsorbent for Simultaneous Removal and Stepwise Recovery of Organic Dyes and Heavy Metal Ions: Fabrication, Performance and Mechanisms. Chem. Eng. J..

[57] Crini G. (2014). Review: A History of Cyclodextrins. Chem. Rev..

[58] Davis M.E., Brewster M.E. (2004). Cyclodextrin-Based Pharmaceutics: Past, Present and Future. Nat. Rev. Drug Discov..

[59] Fenyvesi E., Vikmon M., Szente L. (2016). Cyclodextrins in Food Technology and Human Nutrition: Benefits and Limitations. Crit. Rev. Food Sci. Nutr..

[60] Bender M.L., Komiyama M. (1978). Cyclodextrin Chemistry.

[61] Frömming K.-H., Szejtli J. (1993). Cyclodextrins in Pharmacy.

[62] Sikder M.T., Rahman M.M., Jakariya M., Hosokawa T., Kurasaki M., Saito T. (2019). Remediation of Water Pollution with Native Cyclodextrins and Modified Cyclodextrins: A Comparative Overview and Perspectives. Chem. Eng. J..

[63] Larsen K.L., Endo T., Ueda W.Z.H. (1998). Inclusion complex formation constants of α-, β-, γ-, δ-, -, ζ-, η- and θ-cyclodextrins determined with capillary zone electrophoresis. Carbohydr. Res..

[64] Morin-Crini N., Crini G. (2013). Environmental Applications of Water-Insoluble β-Cyclodextrin–Epichlorohydrin Polymers. Prog. Polym. Sci..

[65] Zhang M., Zhu L., He C., Xu X., Duan Z., Liu S., Song M., Song S., Shi J., Li Y. (2019). Adsorption Performance and Mechanisms of Pb(II), Cd(II), and Mn(II) Removal by a β-Cyclodextrin Derivative. Environ. Sci. Pollut. Res. Int..

[66] Hao Z., Yi Z., Bowen C., Yaxing L., Sheng Z. (2019). Preparing γ-Cyclodextrin-Immobilized Starch and the Study of Its Removal Properties to Dyestuff from Wastewater. Pol. J. Environ. Stud..

[67] Chai K., Ji H. (2012). Dual Functional Adsorption of Benzoic Acid from Wastewater by Biological-Based Chitosan Grafted β-Cyclodextrin. Chem. Eng. J..

[68] Wu D., Hu L., Wang Y., Wei Q., Yan L., Yan T., Li Y., Du B. (2018). EDTA Modified β-Cyclodextrin/Chitosan for Rapid Removal of Pb(II) and Acid Red from Aqueous Solution. J. Colloid Interface Sci..

[69] Zhang H., Li Y.X., Wang P.L., Zhang Y., Cheng B.W., Sun Q.M., Li F. (2019). Synthesis of β-Cyclodextrin Immobilized Starch and Its Application for the Removal of Dyestuff from Waste-Water. J. Polym. Environ..

[70] Jia S., Tang D., Peng J., Sun Z., Yang X. (2019). β-Cyclodextrin Modified Electrospinning Fibers with Good Regeneration for Efficient Temperature-Enhanced Adsorption of Crystal Violet. Carbohydr. Polym..

[71] Chen Y., Ma Y., Lu W., Guo Y., Zhu Y., Lu H., Song Y. (2018). Environmentally Friendly Gelatin/β-Cyclodextrin Composite Fiber Adsorbents for the Efficient Removal of Dyes from Wastewater. Molecules.

[72] Li S., Zhang Y., You Q., Wang Q., Liao G., Wang D. (2018). Highly Efficient Removal of Antibiotics and Dyes from Water by the Modified Carbon Nanofibers Composites with Abundant Mesoporous Structure. Colloids Surf. A Physicochem. Eng. Asp..

[73] Rafatullah M., Sulaiman O., Hashim R., Ahmad A. (2010). Adsorption of methylene blue on low-cost adsorbents: A review. J. Hazard. Mater..

[74] Yuan L., Qiu Z., Yuan L., Tariq M., Lu Y., Yang J., Li Z., Lyu S. (2019). Adsorption and Mechanistic Study for Phosphate Removal by Magnetic Fe3O4-Doped Spent FCC Catalysts Adsorbent. Chemosphere.

[75] Fan L., Zhang Y., Luo C., Lu F., Qiu H., Sun M. (2012). Synthesis and Characterization of Magnetic β-Cyclodextrin-Chitosan Nanoparticles as Nano-Adsorbents for Removal of Methyl Blue. Int. J. Biol. Macromol..

[76] Chen B., Chen S., Zhao H., Liu Y., Long F., Pan X. (2019). A Versatile β-Cyclodextrin and Polyethyleneimine Bi-Functionalized Magnetic Nanoadsorbent for Simultaneous Capture of Methyl Orange and Pb(II) from Complex Wastewater. Chemosphere.

[77] Chen J.Y., Cao S.R., Xi C.X., Chen Y., Li X.L., Zhang L., Wang G.M., Chen Y.L., Chen Z.Q. (2018). A Novel Magnetic β-Cyclodextrin Modified Graphene Oxide Adsorbent with High Recognition Capability for 5 Plant Growth Regulators. Food Chem..

[78] Decher G. (1997). Fuzzy Nanoassemblies: Toward Layered Polymeric. Science.

[79] Whitesides G.M., Grzybowski B. (2002). Self-Assembly at All Scales. Science.

[80] Huang D., Tang Z., Peng Z., Lai C., Zeng G., Zhang C., Xu P., Cheng M., Wan J., Wang R. (2017). Fabrication of Water-Compatible Molecularly Imprinted Polymer Based on β-Cyclodextrin Modified Magnetic Chitosan and Its Application for Selective Removal of Bisphenol A from Aqueous Solution. J. Taiwan Inst. Chem. Eng..

[81] Weiss-Errico M.J., O’Shea K.E. (2019). Enhanced Host–Guest Complexation of Short Chain Perfluoroalkyl Substances with Positively Charged β-Cyclodextrin Derivatives. J. Incl. Phenom. Macrocycl. Chem..

[82] Lin Q., Wu Y., Jiang X., Lin F., Liu X., Lu B. (2019). Removal of Bisphenol A from Aqueous Solution via Host-Guest Interactions Based on Beta-Cyclodextrin Grafted Cellulose Bead. Int. J. Biol. Macromol..

[83] Petitjean M., García-Zubiri I.X., Isasi J.R. (2021). History of cyclodextrin-based polymers in food and pharmacy: A review. Environ. Chem. Lett..

[84] Khalil A.M., Hashem T., Gopalakrishnan A., Schäfer A.I. (2021). Cyclodextrin Composite Nanofiber Membrane: Impact of the Crosslinker Type on Steroid Hormone Micropollutant Removal from Water. ACS Appl. Polym. Mater..

[85] Wang J., Yang F. (2021). Preparation of 2-Hydroxypropyl-β-Cyclodextrin Polymers Crosslinked by Poly(Acrylic Acid) for Efficient Removal of Ibuprofen. Mater. Lett..

[86] Yu T., Xue Z., Zhao X., Chen W., Mu T. (2018). Green Synthesis of Porous β-Cyclodextrin Polymers for Rapid and Efficient Removal of Organic Pollutants and Heavy Metal Ions from Water. New J. Chem..

[87] Usman M., Ahmed A., Yu B., Wang S., Shen Y., Cong H. (2021). Simultaneous Adsorption of Heavy Metals and Organic Dyes by β-Cyclodextrin-Chitosan Based Cross-Linked Adsorbent. Carbohydr. Polym..

[88] Giri A., Sahoo A., Dutta T.K., Patra A. (2020). Cavitand and Molecular Cage-Based Porous Organic Polymers. ACS Omega.

[89] Goel A., Nene S.N. (1995). Modifications in the Phenolphthalein Method for Spectrophotometric Estimation of Beta Cyclodextrin. Starch.

[90] Taguchi K. (1986). Transient Binding Mode of Phenolphthalein-β-Cyclodextrin Complex: An Example of Induced Geometrical Distortion. J. Am. Chem. Soc..

[91] Huang Q., Chai K., Zhou L., Ji H. (2020). A Phenyl-Rich β-Cyclodextrin Porous Crosslinked Polymer for Efficient Removal of Aromatic Pollutants: Insight into Adsorption Performance and Mechanism. Chem. Eng. J..

[92] Figueroa-Lopez K.J., Ortega-Toro R., Villabona-Ortíz Á., Figueroa-Lopez K.J., Ortega-Toro R. (2022). Kinetics and Adsorption Equilibrium in the Removal of Azo-Anionic Dyes by Modified Cellulose. Sustainability.

[93] Sulaiman N.S., Zaini M.A.A., Arsad A. (2019). Evaluation of Dyes Removal by Beta-Cyclodextrin Adsorbent. Mater. Today Proc..

[94] Saifi A., Joseph J.P., Singh A.P., Pal A., Kumar K. (2021). Complexation of an Azo Dye by Cyclodextrins: A Potential Strategy for Water Purification. ACS Omega.

[95] Guo R., Wang R., Yin J., Jiao T., Huang H., Zhao X., Zhang L., Li Q., Zhou J., Peng Q. (2019). Fabrication and Highly Efficient Dye Removal Characterization of Beta-Cyclodextrin-Based Composite Polymer Fibers by Electrospinning. Nanomaterials.

[96] Yang Z., Liu X., Liu X., Wu J., Zhu X., Bai Z., Yu Z. (2021). Preparation of β-Cyclodextrin/Graphene Oxide and Its Adsorption Properties for Methylene Blue. Colloids Surf. B Biointerfaces.

[97] Yadav S., Asthana A., Chakraborty R., Jain B., Singh A.K., Carabineiro S.A.C., Susan M.A.B.H. (2020). Cationic Dye Removal Using Novel Magnetic/Activated Charcoal/β-Cyclodextrin/Alginate Polymer Nanocomposite. Nanomaterials.

[98] Li Y., Zhou Y., Zhou Y., Lei J., Pu S. (2018). Cyclodextrin Modified Filter Paper for Removal of Cationic Dyes/Cu Ions from Aqueous Solutions. Water Sci. Technol..

[99] Wang D., Liu L., Jiang X., Yu J., Chen X. (2015). Adsorption and Removal of Malachite Green from Aqueous Solution Using Magnetic β-Cyclodextrin-Graphene Oxide Nanocomposites as Adsorbents. Colloids Surf. A Physicochem. Eng. Asp..

[100] Li Y., Yu E., Sun S., Liu W., Hu R., Xu L. (2022). Fast and Highly Efficient Adsorption of Cationic Dyes by Phytic Acid Crosslinked β-Cyclodextrin. Carbohydr. Polym..

[101] Anceschi A., Caldera F., Bertasa M., Cecone C., Trotta F., Bracco P., Zanetti M., Malandrino M., Mallon P.E., Scalarone D. (2020). New Poly(β-Cyclodextrin)/Poly(Vinyl Alcohol) Electrospun Sub-Micrometric Fibers and Their Potential Application for Wastewater Treatments. Nanomaterials.

[102] Pedrazzo A.R., Smarra A., Caldera F., Musso G., Dhakar N.K., Cecone C., Hamedi A., Corsi I., Trotta F. (2019). Eco-Friendly β-Cyclodextrin and Linecaps Polymers for the Removal of Heavy Metals. Polymers.

[103] Verma M., Lee I., Sharma S., Kumar R., Kumar V., Kim H. (2021). Simultaneous Removal of Heavy Metals and Ciprofloxacin Micropollutants from Wastewater Using Ethylenediaminetetraacetic Acid-Functionalized β-Cyclodextrin-Chitosan Adsorbent. ACS Omega.

[104] Wang T., He J., Lu J., Zhou Y., Wang Z., Zhou Y. (2021). Adsorptive Removal of PPCPs from Aqueous Solution Using Carbon-Based Composites: A Review. Chin. Chem. Lett..

[105] Hassan M., Naidu R., Du J., Qi F., Ahsan M.A., Liu Y. (2022). Magnetic Responsive Mesoporous Alginate/β-Cyclodextrin Polymer Beads Enhance Selectivity and Adsorption of Heavy Metal Ions. Int. J. Biol. Macromol..

[106] Elbarbary A.M., Bekhit M., El Fadl F.I.A., Sokary R. (2022). Synthesis and Characterization of Magnetically Retrievable Fe3O4/Polyvinylpyrrolidone/Polystyrene Nanocomposite Catalyst for Efficient Catalytic Oxidation Degradation of Dyes Pollutants. J. Inorg. Organomet. Polym. Mater..

[107] Xu L., Zhang M., Wang Y., Wei F. (2021). Highly Effective Adsorption of Antibiotics from Water by Hierarchically Porous Carbon: Effect of Nanoporous Geometry. Environ. Pollut..

[108] Singh G., Lee J.M., Kothandam G., Palanisami T., Al-Muhtaseb A.H., Karakoti A., Yi J., Bolan N., Vinu A. (2021). A Review on the Synthesis and Applications of Nanoporous Carbons for the Removal of Complex Chemical Contaminants. Bull. Chem. Soc. Jpn..

[109] Liu C., Wang P., Liu X., Yi X., Liu D., Zhou Z. (2019). Ultrafast Removal of Cadmium(II) by Green Cyclodextrin Metal–Organic-Framework-Based Nanoporous Carbon: Adsorption Mechanism and Application. Chem. Asian J..

[110] Zhang R., Li Y., Zhu X., Han Q., Zhang T., Liu Y., Zeng K., Zhao C. (2020). Application of β-Cyclodextrin-Modified/PVDF Blend Magnetic Membranes for Direct Metal Ions Removal from Wastewater. J. Inorg. Organomet. Polym. Mater..

[111] He J., Li Y., Wang C., Zhang K., Lin D., Kong L., Liu J., He J., Li Y., Wang C. (2017). Rapid Adsorption of Pb, Cu and Cd from Aqueous Solutions by β-Cyclodextrin Polymers. Appl. Surf. Sci..

[112] Tang P., Sun Q., Zhao L., Tang Y., Liu Y., Pu H., Gan N., Liu Y., Li H. (2019). A Simple and Green Method to Construct Cyclodextrin Polymer for the Effective and Simultaneous Estrogen Pollutant and Metal Removal. Chem. Eng. J..

[113] Yue X., Jiang F., Zhang D., Lin H., Chen Y. (2017). Preparation of Adsorbent Based on Cotton Fiber for Removal of Dyes. Fibers Polym..

[114] Kong L., Yan L., Qu Z., Yan N., Li L. (2015). β-Cyclodextrin Stabilized Magnetic Fe3S4 Nanoparticles for Efficient Removal of Pb(II). J. Mater. Chem. A.

[115] Sun J., Zhao X., Sun G., Zhao H., Yan L., Jiang X., Cui Y. (2022). Phosphate-Crosslinked β-Cyclodextrin Polymer for Highly Efficient Removal of Pb(II) from Acidic Wastewater. New J. Chem..

[116] Peña O.I.G., Zavala M.Á.L., Ruelas H.C. (2021). Pharmaceuticals Market, Consumption Trends and Disease Incidence Are Not Driving the Pharmaceutical Research on Water and Wastewater. Int. J. Environ. Res. Public Health.

[117] Wang L., Zhang D., Xu X., Zhang L. (2016). Application of Ionic Liquid-Based Dispersive Liquid Phase Microextraction for Highly Sensitive Simultaneous Determination of Three Endocrine Disrupting Compounds in Food Packaging. Food Chem..

[118] Deng Z.H., Li N., Jiang H.L., Lin J.M., Zhao R.S. (2019). Pretreatment Techniques and Analytical Methods for Phenolic Endocrine Disrupting Chemicals in Food and Environmental Samples. TrAC—Trends Anal. Chem..

[119] Cortés-Arriagada D. (2021). Elucidating the Co-Transport of Bisphenol A with Polyethylene Terephthalate (PET) Nanoplastics: A Theoretical Study of the Adsorption Mechanism. Environ. Pollut..

[120] Gontard N., Sonesson U., Birkved M., Majone M., Bolzonella D., Celli A., Angellier-Coussy H., Jang G.W., Verniquet A., Broeze J. (2018). A Research Challenge Vision Regarding Management of Agricultural Waste in a Circular Bio-Based Economy. Crit. Rev. Environ. Sci. Technol..

[121] Belenguer-Sapiña C., Pellicer-Castell E., Mauri-Aucejo A.R., Simó-Alfonso E.F., Amorós P. (2020). Cyclodextrins as a Key Piece in Nanostructured Materials: Quantitation and Remediation of Pollutants. Nanomaterials.

[122] Szejtli J. (2010). ChemInform Abstract: Introduction and General Overview of Cyclodextrin Chemistry. ChemInform.

[123] Singh M., Sharma R., Banerjee U.C. (2002). Biotechnological Applications of Cyclodextrins. Biotechnol. Adv..

[124] Plech T., Kapron B., Paneth A., Kosikowska U., Malm A., Strzelczyk A., Staczek P., Swiatek L., Rajtar B., Polz-Dacewicz M. (2015). Search for Factors Affecting Antibacterial Activity and Toxicity of 1,2,4-Triazole-Ciprofloxacin Hybrids. Eur. J. Med. Chem..

[125] Aoki N., Nishikawa M., Hattori K. (2003). Synthesis of Chitosan Derivatives Bearing Cyclodextrin and Adsorption of P-Nonylphenol and Bisphenol A. Carbohydr. Polym..

[126] Wang Z., Zhang B., Fang C., Liu Z., Fang J., Zhu L. (2019). Macroporous Membranes Doped with Micro-Mesoporous β-Cyclodextrin Polymers for Ultrafast Removal of Organic Micropollutants from Water. Carbohydr. Polym..

[127] Gupta V.K., Agarwal S., Sadegh H., Ali G.A.M., Bharti A.K., Makhlouf A.S.H. (2017). Facile Route Synthesis of Novel Graphene Oxide-β-Cyclodextrin Nanocomposite and Its Application as Adsorbent for Removal of Toxic Bisphenol A from the Aqueous Phase. J. Mol. Liq..

[128] Li X., Zhou M., Jia J., Ma J., Jia Q. (2018). Design of a Hyper-Crosslinked Β-Cyclodextrin Porous Polymer for Highly Efficient Removal toward Bisphenol a from Water. Sep. Purif. Technol..

[129] Zhou Y., Cheng G., Chen K., Lu J., Lei J., Pu S. (2019). Adsorptive Removal of Bisphenol A, Chloroxylenol, and Carbamazepine from Water Using a Novel β-Cyclodextrin Polymer. Ecotoxicol. Environ. Saf..

[130] Wang Z., Zhang P., Hu F., Zhao Y., Zhu L. (2017). A Crosslinked β-Cyclodextrin Polymer Used for Rapid Removal of a Broad-Spectrum of Organic Micropollutants from Water. Carbohydr. Polym..

[131] Shi S., Ocampo-Pérez R., Lv J., Liu Q., Nan F., Liu X., Xie S., Feng J. (2021). Diatomite Cross-Linked β-Cyclodextrin Polymers: A Novel Vision of Diatomite Adsorbent for the Removal of Bisphenol A. Environ. Technol. Innov..

[132] Gong T., Zhou Y., Sun L., Liang W., Yang J., Shuang S., Dong C. (2016). Effective Adsorption of Phenolic Pollutants from Water Using β-Cyclodextrin Polymer Functionalized Fe3O4 Magnetic Nanoparticles. RSC Adv..

[133] Lee J.H., Kwak S.Y. (2019). Rapid Adsorption of Bisphenol A from Wastewater by β-Cyclodextrin-Functionalized Mesoporous Magnetic Clusters. Appl. Surf. Sci..

[134] Chen Z.H., Liu Z., Hu J.Q., Cai Q.W., Li X.Y., Wang W., Faraj Y., Ju X.J., Xie R., Chu L.Y. (2020). β-Cyclodextrin-Modified Graphene Oxide Membranes with Large Adsorption Capacity and High Flux for Efficient Removal of Bisphenol A from Water. J. Membr. Sci..

[135] Wang N., Zhou L., Guo J., Ye Q., Lin J.-M., Yuan J., Wang N., Zhou L., Guo J., Ye Q. (2014). Adsorption of Environmental Pollutants Using Magnetic Hybrid Nanoparticles Modified with β-Cyclodextrin. Appl. Surf. Sci..

[136] Pluemsab W., Fukazawa Y., Furuike T., Nodasaka Y., Sakairi N. (2007). Cyclodextrin-Linked Alginate Beads as Supporting Materials for Sphingomonas Cloacae, a Nonylphenol Degrading Bacteria. Bioresour. Technol..

[137] Okoli C.P., Adewuyi G.O., Zhang Q., Diagboya P.N., Guo Q. (2014). Mechanism of Dialkyl Phthalates Removal from Aqueous Solution Using γ-Cyclodextrin and Starch Based Polyurethane Polymer Adsorbents. Carbohydr. Polym..

[138] Cai N., Larese-Casanova P. (2014). Sorption of Carbamazepine by Commercial Graphene Oxides: A Comparative Study with Granular Activated Carbon and Multiwalled Carbon Nanotubes. J. Colloid Interface Sci..

[139] Vernouillet G., Eullaffroy P., Lajeunesse A., Blaise C., Gagné F., Juneau P. (2010). Toxic Effects and Bioaccumulation of Carbamazepine Evaluated by Biomarkers Measured in Organisms of Different Trophic Levels. Chemosphere.

[140] Hamann E., Gruber-Vodicka H., Kleiner M., Tegetmeyer H.E., Riedel D., Littmann S., Chen J., Milucka J., Viehweger B., Becker K.W. (2016). Environmental Breviatea Harbour Mutualistic Arcobacter Epibionts. Nature.

[141] Zhou Q., Fellows A., Flerchinger G.N., Flores A.N. (2019). Examining Interactions Between and Among Predictors of Net Ecosystem Exchange: A Machine Learning Approach in a Semi-Arid Landscape. Sci. Rep..

[142] Jurecska L., Dobosy P., Barkács K., Fenyvesi É., Záray G. (2014). Characterization of Cyclodextrin Containing Nanofilters for Removal of Pharmaceutical Residues. J. Pharm. Biomed. Anal..

[143] Fenyvesi É., Barkács K., Gruiz K., Varga E., Kenyeres I., Záray G., Szente L. (2020). Removal of Hazardous Micropollutants from Treated Wastewater Using Cyclodextrin Bead Polymer—A Pilot Demonstration Case. J. Hazard. Mater..

[144] Feng X., Qiu B., Dang Y., Sun D. (2021). Enhanced Adsorption of Naproxen from Aquatic Environments by β-Cyclodextrin-Immobilized Reduced Graphene Oxide. Chem. Eng. J..

[145] Yadav S., Asthana A., Singh A.K., Chakraborty R., Vidya S.S., Susan M.A.B.H., Carabineiro S.A.C. (2021). Adsorption of Cationic Dyes, Drugs and Metal from Aqueous Solutions Using a Polymer Composite of Magnetic/β-Cyclodextrin/Activated Charcoal/Na Alginate: Isotherm, Kinetics and Regeneration Studies. J. Hazard. Mater..

[146] Duan C., Wang J., Liu Q., Zhou Y., Zhou Y. (2022). Efficient Removal of Salbutamol and Atenolol by an Electronegative Silanized β-Cyclodextrin Adsorbent. Sep. Purif. Technol..

[147] Skwierawska A.M., Nowacka D., Nowicka P., Rosa S., Kozłowska-Tylingo K. (2021). Structural Adaptive, Self-Separating Material for Removing Ibuprofen from Waters and Sewage. Materials.

[148] Zhao F., Repo E., Yin D., Chen L., Kalliola S., Tang J., Iakovleva E., Tam K.C., Sillanpää M. (2017). One-Pot Synthesis of Trifunctional Chitosan-EDTA-β-Cyclodextrin Polymer for Simultaneous Removal of Metals and Organic Micropollutants. Sci. Rep..

[149] Wang R.Q., Wei X.B., Feng Y.Q. (2018). β-Cyclodextrin Covalent Organic Framework for Selective Molecular Adsorption. Chem. Eur. J..

[150] Rahman N., Nasir M. (2020). Effective Removal of Acetaminophen from Aqueous Solution Using Ca (II)-Doped Chitosan/β-Cyclodextrin Composite. J. Mol. Liq..

[151] Rafati L., Ehrampoush M.H., Rafati A.A., Mokhtari M., Mahvi A.H. (2018). Nanocomposite Adsorbent Based on β-Cyclodextrin-PVP-Clay for the Removal of Naproxen from Aqueous Solution: Fixed-Bed Column and Modeling Studies. Desalin. Water Treat..

[152] Dong Z., Tagliavini M., Darmadi J., Trouillet V., Schäfer A.I., Levkin P.A. (2020). Regeneration of β-Cyclodextrin Based Membrane by Photodynamic Disulfide Exchange—Steroid Hormone Removal from Water. Adv. Mater. Interfaces.

[153] Xia J., Zhao P., Zheng K., Lu C., Yin S., Xu H. (2019). Surface Modification Based on Diselenide Dynamic Chemistry: Towards Liquid Motion and Surface Bioconjugation. Angew. Chem. Int. Ed. Engl..

[154] Darwish M., Mohammadi A., Assi N., Abuzerr S., Alahmad Y. (2019). Morphology Selective Construction of β-Cyclodextrin Functionalized Fe3O4-Bi2WO6 Nanocomposite with Superior Adsorptivity and Visible-Light-Driven Catalytic Activity. Front. Chem. Sci. Eng..

[155] Liu X., Yan L., Yin W., Zhou L., Tian G., Shi J., Yang Z., Xiao D., Gu Z., Zhao Y. (2014). A Magnetic Graphene Hybrid Functionalized with Beta-Cyclodextrins for Fast and Efficient Removal of Organic Dyes. J. Mater. Chem. A.

